# Modelling of photosynthesis, respiration, and nutrient yield coefficients in S*cenedemus almeriensis* culture as a function of nitrogen and phosphorus

**DOI:** 10.1007/s00253-021-11484-8

**Published:** 2021-09-14

**Authors:** A. Sánchez Zurano, C. Gómez Serrano, F. G. Acién-Fernández, J. M. Fernández-Sevilla, E. Molina-Grima

**Affiliations:** Chemical Engineering Department, University of Almeria, Ctra. Sacramento, s/n, 04120 Almería, Spain

**Keywords:** Microalgae, Photosynthesis, Respiration, Nitrogen, Phosphorus, Modelling

## Abstract

Photo-respirometric tecniques are applied for evaluating photosynthetic activity in phototrophic organisms. These methods allow to evaluate photosynthetic response under different conditions. In this work, the influence of nutrient availability (nitrate, ammonium, and phosphate) on the photosynthesis and respiration of *Scenedesmus almeriensis* was studied using short photo-respirometric measurements. Both photosynthesis and respiration increasing until saturation value and consecutively diminishing, presenting inhibition by high concentrations. Regarding the influence of phosphorus concentration in microalgae cells, a similar hyperbolic trend was observed but no inhibition was observed at high concentration. Based on these experimental data, the respiration, and the photosynthesis rate of *S. almeriensis* were modelled using Haldane equation for nitrate and ammonium data, and Monod equation for phosphate data. In addition, experiments were performed to determine the yield coefficients for both nitrogen and phosphorus in *S. almeriensis* cultures. The data showed that the nitrogen and phosphorous coefficient yields are not constant, being modified as a function of nutrients concentration, presenting the luxury uptake phenomena. Finally, the proposed models were incorporated into a simulation tool to evaluate the photosynthetic activity and the nutrient yield coefficients of *S. almeriensis* when different culture media and wastewaters are used as a nitrogen and phosphorous source for its growth.

**Key points**

*• Microalgal photosynthesis/respiration vary as a function of nutrients availability.*

*• Photosynthesis inhibition appears at high N-NO*_*3*_^*-*^ *and N-NH*_*4*_^*+*^ *concentrations.*

*• Nutrient yield coefficients are influenced by luxury uptake phenomenon.*

**Graphical abstract:**

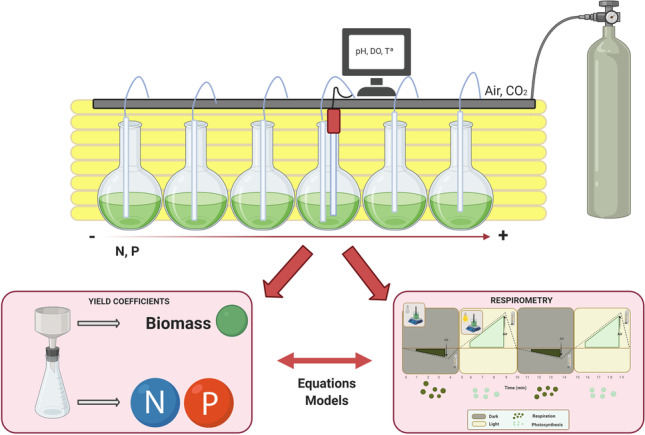

## Introduction

Over the past centuries, CO_2_ concentration in the atmosphere has greatly increased, mainly because of human activities and it leads to known climate change events. Climate change comes along with global consequences on an environmental, social, and economic scale. As a solution to beat these consequences, there is growing interest in developing alternatives for CO_2_ capture, including photosynthetic microorganisms (Aghaalipour et al. 2020). Microalgae cultivation has proposed as a highly promising biological method of CO_2_ because the generated biomass can be used widely (Rodas-Zuluaga et al. 2021). Microalgae biomass have become an eco-friendly alternative in emerging industrial sectors such as aquaculture and animal feed, human nutrition, cosmetics, biofertilizers, and biofuels (Chisti 2008; Acién et al. 2017). However, their large-scale application is still limited by the specific requirements for biomass growth. Microalgae production involves both the maintenance of adequate culture conditions (light, pH, temperature, and dissolved oxygen), related to the reactor design and operating conditions, and the optimal supply of nutrients (carbon, nitrogen, phosphorus, etc.), which affects the production cost (Posten 2009; Acién et al. 2012). An inadequate nutrient supply can greatly reduce the performance of microalgae cells. In general, nutrients usually present in excess, meaning that most processes operate under nutrient-saturation conditions. Nutrients are generally provided as fertilizers to minimize cost; nevertheless, this still represents a relevant contribution to the overall final cost, which ranges from 5 to 20% depending on the production technology (Acién et al. 2012). To reduce the nutrient contribution to the final biomass production cost, utilizing wastewater for microalgae cultivation has been proposed. The advantage of using wastewater as culture medium is that the microalgae can be grown using both organic and inorganic compounds, such as phosphates, ammonium, and nitrates, which are already present in the wastewater, avoiding the cost of nutrients supplementation. At the same time, the wastewaters are treated and can be reused for multiple purposes (Rawat et al. 2011; Acién et al. 2016). Moreover, these sewage treatment systems based on microalgae can be optimized by an adequate CO_2_ supplementation, which allow to obtain high biomass productivity and nutrients removal (Molino et al. 2019).

Several works have revealed the robustness of microalgae-based wastewater systems in terms of biomass productivity and the high contaminant removal rates, the focus being on developing mathematical models capable of simulating and optimizing microalgae wastewater treatment. Although the first microalgae models were based on single factors, such as light intensity (Molina-Grima et al. 1994), nitrogen (Smit 2002), or phosphorus (Sommer 1991), the current models have introduced multiple factors affecting microalgae performance such as irradiance, temperature, pH, and dissolved oxygen (Costache et al. 2013; Ippoliti et al. 2016). However, the use of wastewater for microalgae production involves not only microalgae performance but also different bacterial populations appear in these systems, which increases the complexity of the mechanistic models (Solimeno et al. 2015). Various types of mathematical models have been developed for understanding the interaction between the microalgae and the bacteria. Since Buhr and Miller (1983) developed the first mathematical model to describe microalgae and bacteria growth in wastewater, multiple microalgae-bacteria models for wastewater treatment have been proposed and validated (Reichert and Vanrolleghem 2001; Sah et al. 2020; Solimeno et al. 2019, 2017; Wágner et al. 2016; Zambrano et al. 2016).

Many microalgae-bacteria models have been validated in terms of the influence of nutrient availability on microalgae/bacteria consortia performance, and considerable knowledge has been accrued regarding the behavior of both heterotrophic and nitrifying bacteria as a function of nutrient concentration. Nonetheless, the performance of microalgae cells has hardly been studied. For instance, the mechanistic models (ASM1, ASM2, ASM2D, and ASM3) of the Activated Sludge Model (ASM) series, promoted by the International Water Association, already consider the influence of organic carbon sources, ammonium, nitrate, and phosphorus on bacterial performance—the variation in growth rates based on the concentration of the respective nutrients fitting the Monod model, with constant coefficient yields being determined for each microorganism type and nutrient type (Gernaey et al. 2004; M Henze et al. 2015). Of the scarce information available regarding microalgae performance, BIOALGAE is one of the most nutrient-complete models (Solimeno et al. 2017). Most papers in the literature provide information on experiments carried out under excess nutrient conditions, focusing on maximizing the microalgal cell performance. Conversely, other papers looking at nutrient limitation conditions focus on the kinetics of secondary metabolite accumulation. However, little information is available regarding the influence of nutrient concentration on microalgal cell performance (Fernandes et al. 2016; Mc Gee et al. 2020).

For the bacteria characterization of activated sludge, respirometric techniques have been applied as a rapid tool to ascertain kinetic growth parameters (Ellis et al. 1996). Over recent years, this respirometry, which has traditionally been applied to bacteria in wastewater, has been extended to phototrophic cultures. In algal cultures, the use of respirometry allows one to determine the phototrophic activity by measuring the oxygen production rate (*OPR*) under light conditions and the oxygen uptake rate (OUR) in the dark. These measurements, which are based on oxygen production/consumption, are rapid and easily obtainable (Tang et al. 2014; Sánchez-Zurano et al. 2020). In fact, respirometric methods have been evaluated and applied to photosynthetic cultures for biokinetic parameter determination (Decostere et al. 2013). This methodology allows one to determine the effect of culture parameters on microalgae activity and to measure kinetic parameters such as the nutrients’ half-saturation constants, thus avoiding batch experiments, which are very time consuming (> 10 days); in addition, the results might be affected by biomass debris formation (Robertson et al. 1998; Sforza et al. 2019).

In this work, a photo-respirometric method is proposed as a simple, innovative, and rapid method to measure kinetic parameters in microalgae cultures. Respirometry was applied to measure the nutrient saturation coefficients of *Scenedesmus almeriensis*, relating to the main nutrients present in the wastewater (nitrate, ammonium, and phosphate). The respirometric experiments allow to determine the kinetic parameters of the net photosynthesis rate and the net respiration rate under autotrophic conditions. Experiments were also performed to determine the coefficient yields, both for nitrogen and phosphorus, in *S. almeriensis* cultures. This study allowed an in-depth analysis of the importance of adequate nutrient supplementation in the microalgae cultivation. All the obtained parameters allow to increase the understanding of the effect of nutrients on microalgae-based processes and to improve the current mechanistic models for microalgae-bacteria systems.

## Materials and methods

### Microalgal species and culture conditions

The microalga *S. almeriensis* CCAP 276/24 was obtained from the culture collection of the Department of Chemical Engineering of the University of Almería. The inoculum of this strain was grown photoautotrophically in a Erlenmeyer spherical flask (1.0 l capacity) and inoculated weekly with fresh modified Arnon medium (Allen and Arnon 1955) (Table 1). The culture was continuously supplied with an air–1%CO_2_ mixture to control the pH at 8.0. The Erlenmeyer spherical flask was maintained at 24 °C, controlled by regulating the air temperature in the chamber. The culture was artificially illuminated on a 12:12 h L/D cycle using four Philips PL-32 W/840/4p white-light lamps, providing an irradiance of 750 μE/m^2^ s on the spherical 1.0 L flask surface.

**Table 1:** Average composition of the modified Arnon medium. Concentrations expressed as mg·L^−1^

Parameters	Arnon
pH	7.5 ± 0.2
COD	16.0 ± 1.2
Sulphate	6.3 ± 0.8
Nitrogen-nitrate	140.0 ± 4.5
Chloride	78.9 ± 2.1
Sodium	276.1 ± 7.9
Potassium	325.1 ± 6.3
Calcium	364.9 ± 5.5
Magnesium	12.2 ± 0.6
Phosphorus-phosphate	39.3 ± 3.1
Nitrogen-ammonium	0.0 ± 0.1
Iron	5.0 ± 0.3
Copper	0.02 ± 0.0
Manganese	0.5 ± 0.02
Zinc	0.06 ± 0.01
Boron	0.4 ± 0.03
TC	52.4 ± 4.9
TN	140.0 ± 4.5
TP	39.3 ± 3.1

Values correspond to the mean ± SD

### Experimental set-up

To evaluate the oxygen production/consumption rates of *S. almeriensis* as a function of nutrient availability, experiments were performed in Erlenmeyer spherical flasks (1.0 L capacity) filled to 650 mL with Arnon medium, modified according to the specific assay, and 20% of *S. almeriensis* inoculum. To study the effect of the concentration of each main nutrient (nitrogen and phosphorous), the other one was maintained in the same concentration that established Arnon medium. Moreover, the rest of the minor and major nutrients were kept as defined the protocol. Three sets of experiments were performed: (i) at different nitrate concentrations from 0 to 200 mgN·L^.1^, maintaining of phosphate at the concentration that indicate Arnon medium, (ii) without nitrate but using ammonium as a nitrogen source, at different concentrations from 0 to 200 mgN·L^−1^, and (iii) at different phosphate concentrations from 0 to 30 mgP·L^−1^, maintaining nitrogen in form of nitrate as a nitrogen source (at the concentration that indicate Arnon medium). The modified Arnon mediums were sterilized in an autoclave at 120 °C for 20 min. The Erlenmeyer spherical flasks were operated in batch mode to take samples for the respirometric tests and nutrient yield coefficient determination. Each reactor was aerated at a rate of 0.2 v/v/min with CO_2_ injected on demand (pH = 8). The reactors were continuously illuminated artificially using eight 28 W fluorescent tubes (Philips Daylight T5), providing an irradiance of 1350 μE/m^2^ s on the spherical 1.0 L flask surface.

### Respirometric measurements

To determine the oxygen production rate and oxygen consumption rate of *S. almeriensis*, a photo-respirometer was used. This device allows one to measure the variation in the dissolved oxygen concentration in microalgae samples under different conditions. The oxygen measurements were performed in a jacketed 60 mL glass flask which was mixed by a magnetic stirrer. The glass flask was artificially illuminated using two controlled LED lamps situated to the right and left of the flask. The desired irradiance inside the flask could be automatically controlled. The dissolved oxygen concentration in the microalgae samples was continuously measured by a sensor (Crison 5002, Barcelona, Spain) located inside the glass flask. There were also sensors for temperature, pH, and irradiance placed within the flask. As the temperature was controlled at 24 °C, the temperature effect was disregarded in the growth kinetic parameters. The reliability of this method was highlighted by (Sánchez-Zurano et al. 2020), since the authors proposed a standardization of the photo-respirometry method, defining a protocol to follow, the biomass concentration and irradiance used during the measurements, and the oxygen mass transfer coefficient (K_L_a) used to correct the influence of oxygen desorption on the photo-respirometric measurements (Sánchez-Zurano et al. 2020).

The influence of the oxygen desorption on the respirometric measurements was corrected using the oxygen mass transfer coefficient (${{\varvec{K}}}_{{\varvec{L}}}{\varvec{a}})$. This value was determined in absence of aeration experimentally. The method used consisted in measuring the dissolved oxygen concentration versus time profiles in the same chemical-physical conditions applied during the respirometric tests. For this, a cell-free sample was placed in the measurement device and the concentration of oxygen was increased to 130%Sat by bubbling with the pure O_2_ gas. After this, the bubbling was stopped and the variation in oxygen concentration (${{\varvec{C}}}_{{\varvec{O}}2}$) with time was monitored for around 4 h. The (${{\varvec{K}}}_{{\varvec{L}}}{\varvec{a}})$ in the system quantifies the proportionality between the oxygen exchange between the liquid and gas phases and the driving force expressed as (${C}_{02}^{*}-{C}_{O2}$) leading to the following elementary mass balance:

1 $$\frac{d\mathrm{CO}2}{dt}={K}_{L}a \left({C}_{02}^{*}-{C}_{O2}\right)$$

The determination of the ${{\varvec{K}}}_{{\varvec{L}}}{\varvec{a}}$ is described in details by Sánchez-Zurano et al. 2020. The ${{\varvec{K}}}_{{\varvec{L}}}{\varvec{a}}$ value obtained was 1.08 h^−1^.

**Fig. 1:**
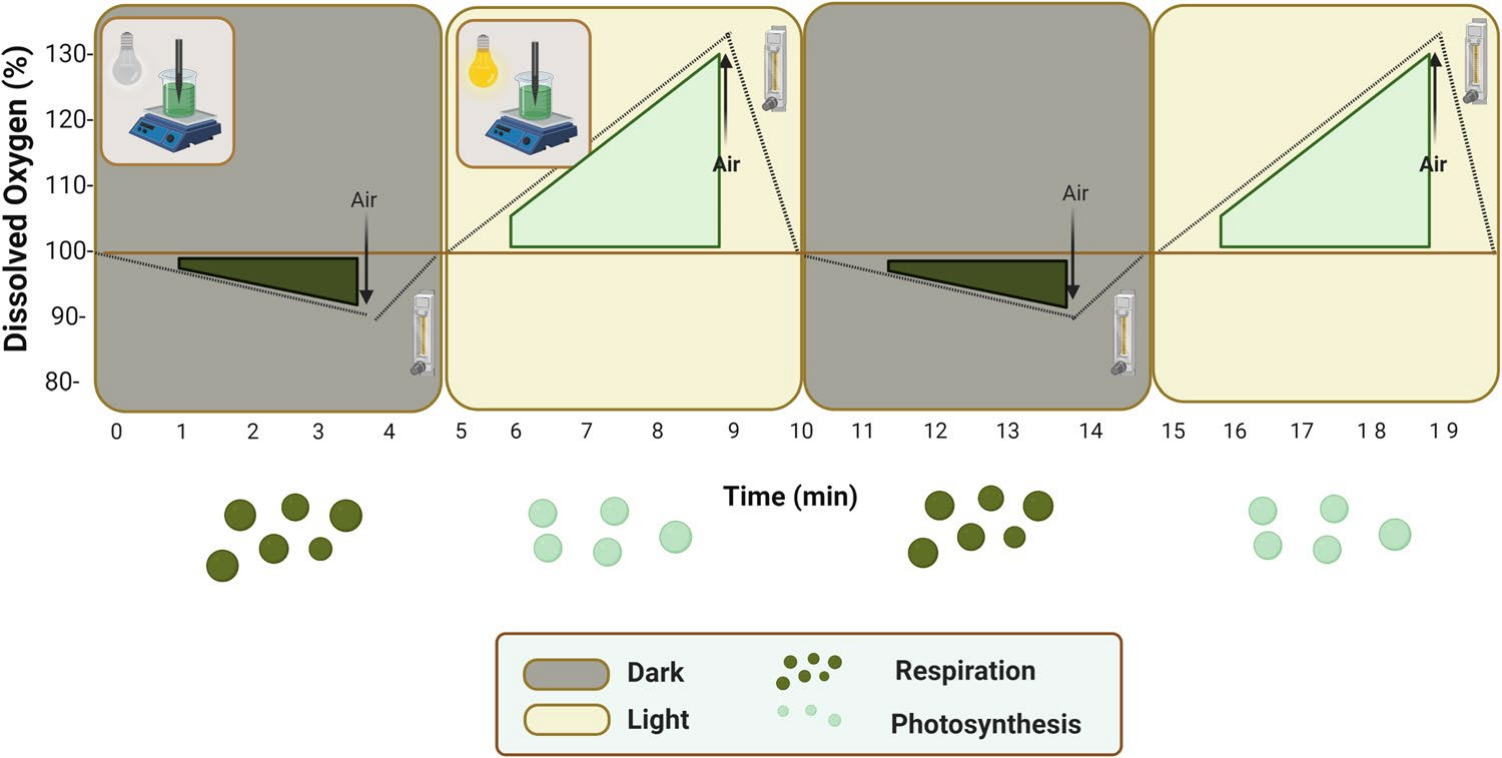
Typical result of a respirometric test. Dark and light phases are reported together with the addition of air to recover 100% dissolved oxygen

The protocol proposed relies on the measurement of oxygen produced or consumed by microalgal biomass under different nitrogen and phosphorous concentrations. The procedure proposed is based on the oxygen production/consumption under cycles of light and dark as a function of a single variable at a time, while keeping the other variable constant. These produced/consumed oxygen measurements allow us to determine the net photosynthesis rate and the net respiration rate, respectively. The methodology consists of inoculating the Erlenmeyer spherical flaks at different stages with different concentrations of the studied variable and waiting 30 min for acclimatization. After that time, samples of each microalgae culture were taken to measure the oxygen production during the light phases and the oxygen consumption during the dark phases (Fig. 1). Each culture sample was placed inside the photo-respirometer and then exposed to light–dark cycles of 4 min each to measure and record the variation in dissolved oxygen under each condition (Fig. 1). The first minute of exposure was disregarded as it was considered an adaptation time. Between the dark and light periods, air was provided to recover the 100%Sat of the dissolved oxygen. During light periods, oxygen generation is expected as a result of the active photosynthesis carried out by the microalgae whereas during the dark periods, oxygen is consumed by the endogenous respiration rate. The microalgae’s oxygen production rate (*OPR*) was calculated from the slope of the dissolved oxygen concentration over the last 3 min of the light phases $\left(\frac{d{\left[O2\right]}_{L}}{dt}\right)$, dividing by the biomass concentration $(Cb)$ (Eq. 2).

2 $$OPR=\frac{1}{Cb}\left(\frac{d{\left[O2\right]}_{L}}{dt}\right)$$

Similarly, the oxygen consumption rate (*OCR*) was calculated from the slope of the dissolved oxygen concentration over the last 3 min of the dark phases $\left(\frac{d{\left[O2\right]}_{D}}{dt}\right)$, dividing by the biomass concentration $(Cb)$ (Eq. 3).

3 $$OCR=\frac{1}{Cb}\left(\frac{d{\left[O2\right]}_{D}}{dt}\right)$$

Finally, the net photosynthesis rate (*NPR*) was calculated as the difference between the oxygen production rate and the oxygen consumption rate (Eq. 4). In addition, the microalgae respiration rate (*MRR*) was defined as the oxygen consumption rate (Eq. 5).

4 $$NPR=OPR-OCR$$

5 $$MRR=OCR$$

The maximal photosynthetic and respiratory activities, measured under an increasing nutrient concentration, were used to normalize the experimental data obtained from 0 to 1. Each *OPR* and *OCR* value was estimated as the average of at least four measurements (i.e., four dark–light cycles of 4:4 min each).

### Estimation of the nutrient yield coefficients

The coefficient yield for the macronutrients (nitrogen and phosphorus) was determined as the variation of the substrate to biomass concentration ratio; that is to say, the coefficient yield was defined as the amount of substrate consumed over the amount of microalgae produced. Determining these coefficients is mandatory for optimizing the mathematical models which simulate the biomass growth and the nutrient removal in microalgal processes. The nitrogen/biomass yield and phosphorous/biomass yield were expressed in g N/g dry biomass and g P/g dry biomass, respectively.

For this purpose, samples from each spherical glass flask containing the different concentrations of nitrogen and phosphorus were taken over 24 h to determine the biomass concentration by the dry weight and to measure the nutrients in the sample’s supernatant.

### Biomass concentration and analytical methods

The biomass concentration (Cb) was measured by dry weight. Aliquots containing 100 mL of the culture were filtered through the Macherey–Nagel MN 85/90 glass fiber filters. Then, the filters were dried in an oven at 80 °C for 24 h. Standard official methods were used to analyze the composition of the wastewater samples and the water from the reactors. The phosphate was measured by visible spectrophotometry through the phospho-vanado-molybdate complex (Phosphate Standard for IC: 38,364). The nitrate was quantified by measuring optical density at 220 nm and 275 nm (Nitrate Standard for IC: 74,246). The ammonium was measured according to the Nessler method (Ammonium standard for IC: 59,755).

### Software and statistical analysis

The DaqFactory data acquisition and control software (Azeotech, USA) were used to gather the photosynthesis and respiration rate data. All the measurements were performed in triplicate (at least) to allow us to calculate the mean values and standard deviations shown. Data analysis was carried out using the Statgraphics Centurion XVI software package, in which non-linear regression was used to fit experimental data to the proposed models, and to determine the characteristic parameter values. These models were used to obtain simulations in Microsoft Excel.

## Results

### Influence of the nutrient concentration on the photosynthesis and respiration rates

**Fig. 2:**
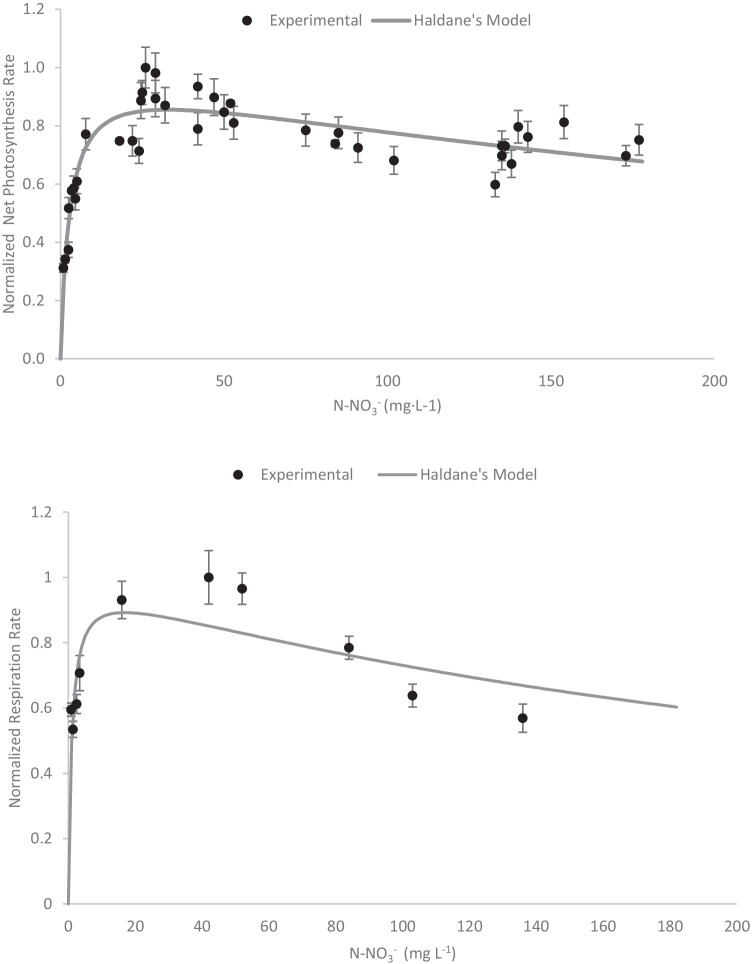
Influence of nitrogen in form of nitrate on the normalized photosynthesis rate of *S. almeriensis* (A) and on the normalized respiration rate of *S. almeriensis* (B). Lines correspond to the fit of the proposed models (Eq. 6, Eq. 7)

To study the influence of nitrate on *S. almeriensis* performance, concentrations ranging from 0 to 200 mgN·L^−1^ were assayed, which correspond to a nitrate range from 0 to 900 mgNitrate·L^−1^. Experiments performed in which the nitrogen in form of nitrate concentration in the culture medium was modified have shown that both the net photosynthesis rate and the net respiration rate increase hyperbolically with the nitrogen concentration, achieving a maximum value in the 20–40 mgN·L^−1^ range; above this value, both the net photosynthesis rate and the net respiration rate decrease (Fig. 2). According to these figures, inhibition by nitrate does take place, even at moderate concentrations of 200 mgN-NO_3_^−^·L^−1^ (approximately 40 mgN·L^−1^); this has not been widely reported. Data processing was subsequently carried out to calculate the normalized maximum net photosynthesis and respiration rates, being 130 and 25 mgO2·g_biomass_^−1^·h^−1^ for the specific maximum photosynthetic rate (PO_2,max_) and the specific maximum respiration rate (RO_2,max_), respectively. Experimental data have been fitted to a model which considers inhibition by substrate, such as the Haldane equation (Eq. 6) (Armstrong 1930), in which the net photosynthesis (PO_2_) rate is a function of the nitrogen concentration (N-NO_3_^−^), the nitrogen half-saturation constant (*K*_S,N-NO3_^−^), and the inhibition parameter constant (*K*_I_). By fitting experimental data to this equation, the characteristic parameter values were determined (*K*_S,N-NO3_^−^ = 2.77 mgN-NO_3_^−^·L^−1^ and *K*_I,N-NO3_^−^ = 279 mgN-NO_3_^−^·L^−1^), verifying that the model reproduces the behavior of the measurements performed.

6 $$\overline{{PO }_{2}(\left[N-{NO}_{3}^{-}\right])}=\frac{\left[N-{NO}_{3}^{-}\right]}{\left[N-{NO}_{3}^{-}\right]+ {K}_{S,N-NO3-}+\frac{{\left[N-{NO}_{3}^{-}\right]}^{2}}{{K}_{I,N-NO3-}}}$$

Concerning the respiration rate, which was determined by oxygen measurements in the dark, the data also show a pattern of inhibition by substrate, The respiration rate is zero at a null nitrogen in form of nitrate concentration but increases with the concentration to reach a maximum at 20 mgN·L^−1^ (approximately 90 mgNitrate·L^−1^); it then decreases at higher nitrogen concentrations. The data have also been fitted to the Haldane equation (Eq. 7). The characteristic parameter values obtained were as follows: *K*_R, N-NO3_^−^ = 1.02 mgN-NO_3_^−^·L^−1^ and *K*_I,R,N-NO3_^−^ = 279 mg N-NO_3_^−^·L^−1^. The results show that the selected microalgae only need low nitrogen concentrations to perform the photosynthesis and respiration properly.

7 $$\overline{{RO }_{2}(\left[N-{NO}_{3}^{-}\right])}=\frac{\left[N-{NO}_{3}^{-}\right]}{\left[N-{NO}_{3}^{-}\right]+ {K}_{R,N-NO3-}+\frac{{\left[N-{NO}_{3}^{-}\right]}^{2}}{{K}_{I,R,N-NO3-}}}$$

**Fig. 3:**
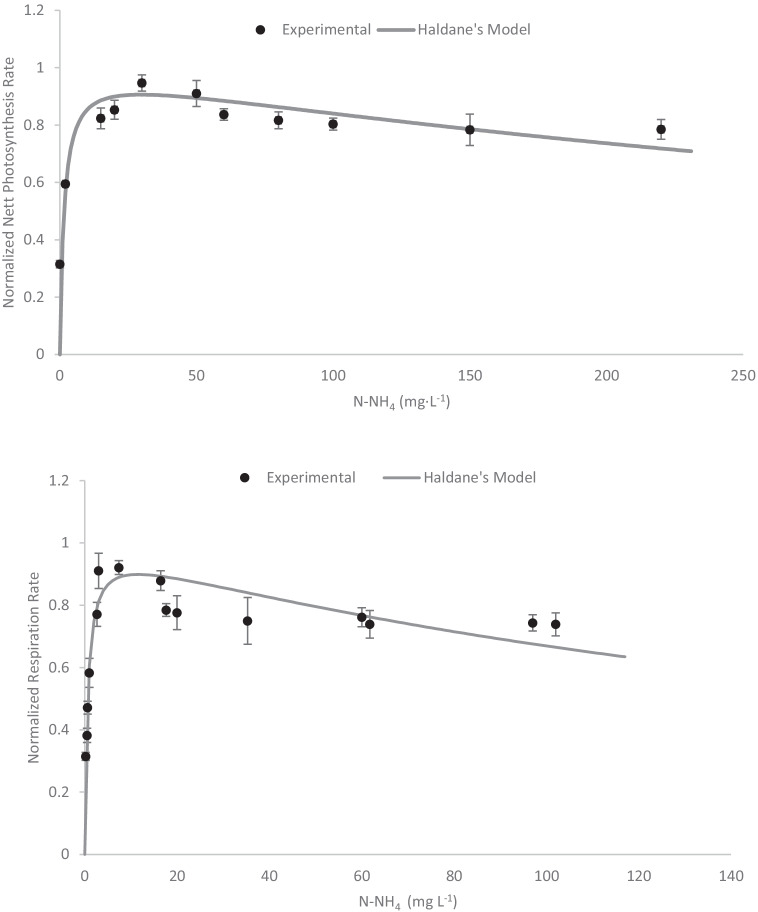
Influence of nitrogen in form of ammonium on the normalized photosynthesis rate of *S. almeriensis* (A) and on the normalized respiration rate of *S. almeriensis* (B). Lines correspond to the fit of the proposed models (Eq. 8, Eq. 9)

To determine the behavior of *S. almeriensis* with respect to N-NH_4_^+^, experiments were performed at concentrations ranging from 0 to 250 mgN·L^−1^, which corresponds to ammonium range of 0 to 320 mg N-NH_4_^+^ L^−1^ (Fig. 3). The results showed a similar trend as previously found with nitrate both the net photosynthesis rate and the net respiration rate increased along with the N-NH_4_^+^ concentration until a value of 10–20 mg N-NH_4_^+^ L^−1^ was reached; above this value, both the net photosynthesis rate and the net respiration rate decreased. As before, a model considering the existence of inhibition by substrate has been used to fit the experimental results. These experimental data were modelled using the Haldane equation (Eq. 8, Eq. 9), in which the characteristic parameter values for the net photosynthesis rate (PO_2_) were determined (*K*_S,N-NH4_^+^  = 1.54 mgN-NH_4_^+^·L^−1^ and *K*_I,N-NH4_ = 571 mgN-NH_4_^+^), verifying that the model reproduces the behavior indicated by the measurements. For the respiration rate (RO_2_), the kinetic parameters for the ammonium concentrations were calculated (*K*_R, N-NH4_^+^ = 0.65 mgN-NH_4_^+^·L^−1^ and *K*_I,R,N-NH4_ = 205 mgN-NH_4_^+^·L^−1^).

8 $$\overline{{PO }_{2}(\left[N-{NH}_{4}^{+}\right])}=\frac{\left[N-{NH}_{4}^{+}\right]}{\left[N-{NH}_{4}^{+}\right]+ {K}_{S,N-NH4+}+\frac{{\left[N-{NH}_{4}^{+}\right]}^{2}}{{K}_{I,N-NH4+}}}$$

9 $$\overline{{RO }_{2}(\left[N-{NH}_{4}^{+}\right])}=\frac{\left[N-{NH}_{4}^{+}\right]}{\left[N-{NH}_{4}^{+}\right]+ {K}_{R,N-NH4+}+\frac{{\left[N-{NH}_{4}^{+}\right]}^{2}}{{K}_{I,R,N-NH4+}}}$$

**Fig. 4:**
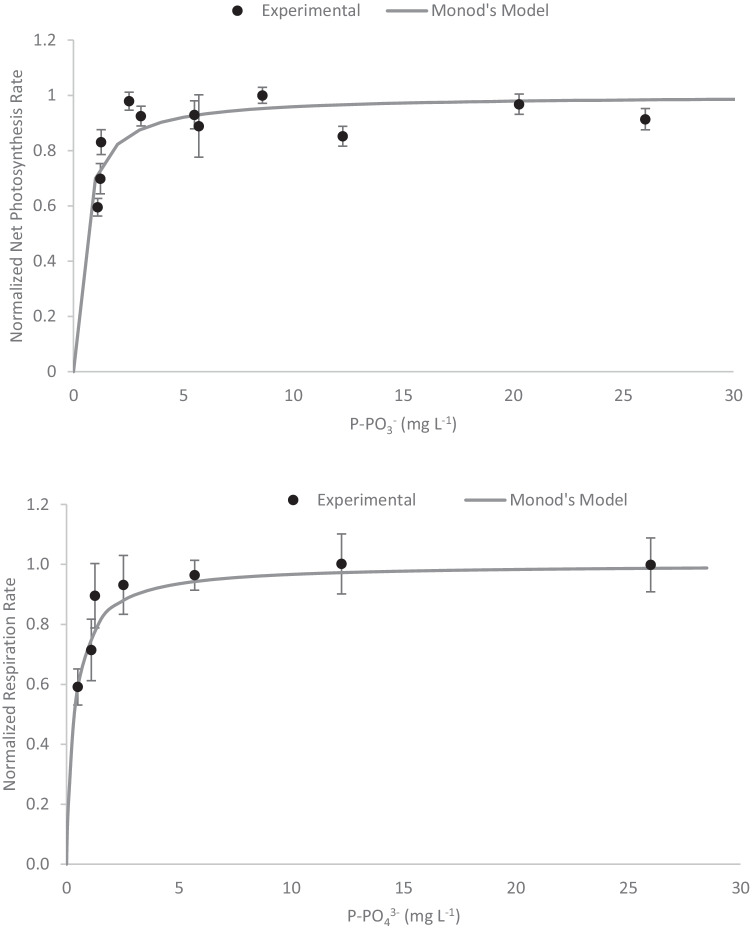
Influence of phosphorus on the normalized photosynthesis rate of *S. almeriensis* (A) and on the normalized respiration rate of *S. almeriensis* (B). Lines correspond to the fit of the proposed models (Eq. 10)

Concerning to the phosphorous, in this work, the experiments were performed up to a concentration of 120 mg PO_4_^3−^·L^−1^, which corresponds to 40 mg P-PO_4_^3−^·L^−1^. The results showed that the net photosynthesis and respiration rates hyperbolically increased with the phosphorous concentration in the concentration range assayed, with no inhibition being observed at higher concentrations (Fig. 4). To fit the experimental data, the Monod model has been used (Eq. 10), in which the characteristic parameter values for the net photosynthesis rate and the net respiration rate were determined (*K*_S,P-PO4_ = 0.43 mg P-PO_4_^3−^·L^−1^ and *K*_R, P-PO4_ = 0.35 mg P-PO_4_^3−^·L^−1^).

10 $$\overline{{PO }_{2}(\left[P-{PO}_{4}^{3-}\right])}=\frac{\left[P-{PO}_{4}^{3-}\right]}{\left[P-{PO}_{4}^{3-}\right]+ {K}_{S,P-PO4}}$$

11 $$\overline{{RO }_{2}(\left[P-{PO}_{4}^{3-}\right])}=\frac{\left[P-{PO}_{4}^{3-}\right]}{\left[P-{PO}_{4}^{3-}\right]+ {K}_{R,P-PO4}}$$

In summary, the values obtained for all the characteristic parameters are shown in Table 2.

**Table 2:** Values for the proposed model’s parameter characteristics and confidence intervals

Nitrate models			Ammonium models			Phosphate models		
Parameter	Value	Units	Parameter	Value	Units	Parameter	Value	Units
*K*_S,N-NO3_^−^	2.77 ± 0.28	mgN-NO_3_^−^·L^−1^	*K*_S,N-NH4_^+^	1.54 ± 0.15	mgN-NH_4_^+^·L^−1^	*K*_S, P-PO4_	0.43 ± 0.06	mg P-PO_4_^3−^·L^−1^
*K*_I,N-NO3_^−^	386.6 ± 42.5	mgN-NO_3_^−^·L^−1^	*K*_I,N-NH4_^+^	571 ± 49.2	mgN-NH_4_^+^·L^−1^	*K*_R, P-PO4_	0.35 ± 0.03	mg P-PO_4_^3−^·L^−1^
*K*_R,N-NO3_^−^	1.02 ± 0.12	mgN-NO_3_^−^·L^−1^	*K*_R,N-NH4_^+^	0.65 ± 0.08	mgN-NH_4_^+^·L^−1^			
*K*_I,R,N-NO3_^−^	279 ± 25.4	mgN-NO_3_^−^·L^−1^	*K*_I,R,N-NH4_^+^	205 ± 21.3	mgN-NH_4_^+^·L^−1^			

### Influence of nutrient concentration on the yield coefficients

Once the influence of the nutrient concentrations on the photosynthesis and respiration rates of *S. almeriensis* cells had been determined, experiments were also performed to determine the yield coefficients. Experiments were performed under the same conditions as before, in the same concentration ranges, to determine if nutrient concentrations influence the coefficient yield values.

**Fig. 5:**
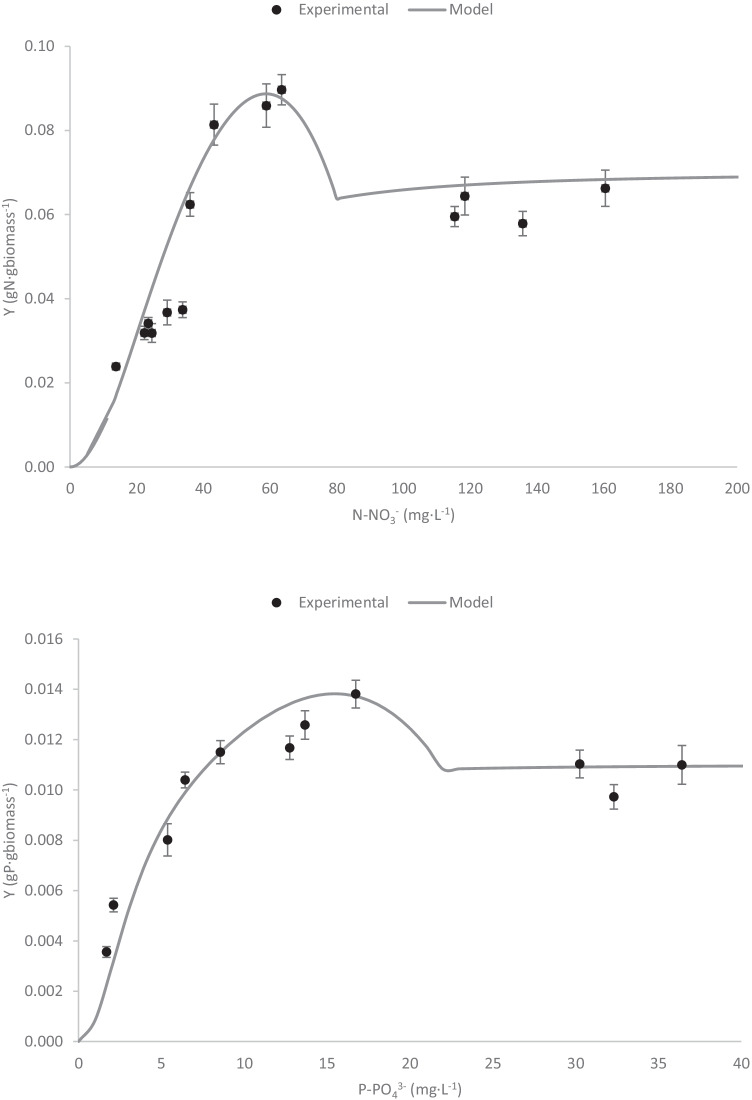
Nutrient yield coefficients of *S. almeriensis*: nitrogen yield coefficient (A); phosphorous yield coefficient (B). Lines correspond to the fit of the proposed models (Eq. (11), Eq. (12))

The data show that the nitrogen and phosphorous coefficient yields are not constant, being modified as a function of the nutrient’s concentration (Fig. 5). The results show that the nitrogen and phosphorous coefficient yields increase as nitrogen or phosphorus increase in the culture medium, observing a peak at 70 mgN-NO_3_^−^·L^−1^ and 18 mgP-PO_4_^3−^·L^−1^, respectively. Modelling this phenomenon is complex, since if the trend of the experimental data is considered, one might think that a certain inhibition appears in the yield coefficients. However, it would not be an inhibition, but the data show a variability in the value of the yield coefficients due to its relationship with the concentration of nitrogen and phosphorus in the medium. To model this phenomenon, the sum of two equations has been applied—the hyperbolic equation and the cardinal equation. The former, which is typically used for microbial growth kinetics, has been used to explain the increase in the nitrogen and phosphorous coefficient yields as the nitrogen or phosphorous concentrations increase in the medium. In addition, to describe the peaks observed both in the nitrogen and phosphorous coefficient yields, the cardinal equation has been applied within the minimum and maximum ranges established. The cardinal model allows one to define the maximal, minimal, and optimal conditions for whichever variable, fitting its influence into the biological system performance as a Gaussian function (Bernard and Rémond 2012). Using the cardinal equation allows one to obtain the “optimal nutrient concentration value” in which the nitrogen and phosphorous yield coefficients are higher. Regarding nitrogen, the coefficient values obtained ranged from 0.02 to 0.09 gN-NO_3_^−^·g_biomass_^−1^. Concerning the phosphorus, the results showed that the phosphorous yield coefficient ranged from 0.004 to 0.014 gP-PO_4_^3−^·g_biomass_^−1^ at the phosphorous concentrations tested. Subsequently, the nitrogen and phosphorous yield coefficients were fitted to the sum of the hyperbolic and cardinal models (Eq. 12, Eq. 13) from which the characteristic parameter values for the nitrogen yield coefficient (*Y*_gN/gbiomass, max_ = 0.07 gN-NO_3_^−^·g_biomass_^−1^, *K*_S,YN_ = 25 mgN-NO_3_^−^·L^−1^, *m* = 2, *N*_max_ = 80 mgN-NO_3_^−^·L^−1^, *N*_min_ = 10 mgN-NO_3_^−^·L^−1^, *N*_opt_ = 55 mgN-NO_3_^−^·L^−1^) and phosphorous yield coefficient (*Y*_gP/gbiomass, max_ = 0.011 gP-PO_4_^3−^·g_biomass_^−1^, *K*_S,YP_ = 3.2 mgP-PO_4_^3−^·L^−1^, *m* = 2.14, P_max_ = 22 mgP-PO_4_^3−^·L^−1^, *P*_min_ = 2 mgP-PO_4_^3−^·L^−1^, *P*_opt_ = 15 mgP-PO_4_^3−^·L^−1^) were determined (Table 3).

**Table 3:** Values for nitrogen and phosphorous yield and confidence intervals

Nitrogen yield model			Phosphorous yield model		
Parameter	Value	Units	Parameter	Value	Units
*Y*_gN/gbiomass, max_	0.07 ± 0.008	g N-NO_3_^−^·g_biomass_^−1^	*Y*_gP/gbiomass, max_	0.011 ± 0.001	gP-PO_4_^3−^·g_biomass_^−1^
*K*_S, YN_	25 ± 2.7	mg N-NO_3_^−^·L^−1^	*K*_S, YP_	3.2 ± 0.34	mg P-PO_4_^3−^··L^−1^
*m*	2 ± 0.2	-	*m*	2.14 ± 0.22	-
*N*_max_	80 ± 7.2	mg N-NO_3_^−^·L^−1^	*P*_max_	22 ± 2.3	mg P-PO_4_^3−^·L^−1^
*N*_min_	10 ± 0.9	mg N-NO_3_^−^·L^−1^	*P*_min_	2 ± 0.3	mg P-PO_4_^3−^··L^−1^
*N*_opt_	55 ± 4.9	mg N-NO_3_^−^·L^−1^	*P*_opt_	15 ± 1.7	mg P-PO_4_^3−^··L^−1^

12 $${Y}_{N/ biomass}=\left[\frac{Y_{N/ biomass,\mathit{\max}}\cdot {\left[N\right]}^m}{{\left[N\right]}^m+{K_{S, YN}}^m\ }\right]+\left[\frac{\left(N- Nmax\right)\left(N- Nmin\right)2}{\left( Nopt- Nmin\right)\left(\left(\left( Nopt- Nmin\right)\left(N- Nopt\right)\right)-\left(\left( Nopt- Nmax\right)\left( Nopt+ Nmin-2N\right)\right)\right)}\right.$$

13 $${Y}_{P/ biomass}=\left[\frac{Y_{P/ biomass,\mathit{\max}}\cdot {\left[P\right]}^m}{{\left[P\right]}^m+{K_{S, YP}}^m\ }\right]+\left[\frac{\left(P- Pmax\right)\left(P- Pmin\right)2}{\left( Popt- Pmin\right)\left(\left(\left( Popt- Pmin\right)\left(P- Popt\right)\right)-\left(\left( Popt- Pmax\right)\left( Popt+ Pmin-2P\right)\right)\right)}\right.$$

### Performance of S. almeriensis cells as a function of the culture medium

Once the effects of nitrogen and phosphorus were evaluated and modelled, both for the photosynthesis rate and for the respiration rate, simulations were performed to determine the performance of *S. almeriensis* cells as a function of the culture medium used to produce them. These simulations were performed mainly considering the culture media, from the standard culture medium prepared using fertilizers to the different wastewater types, even including wastewater that had been depurated in accordance with the regulations. Wastewater that has already been treated should contain a low nutrient concentration (5–10 mg-N·L^−1^ and 1–2 mg-N·L^−1^). In this work, we considered two possibilities: treated wastewater with the maximum nutrient concentration for safe disposal (10 mg-N·L^−1^) and treated wastewater complying to the new limits (5 mg-N·L^−1^) (European Directive 91/271/CEE).

**Fig. 6:**
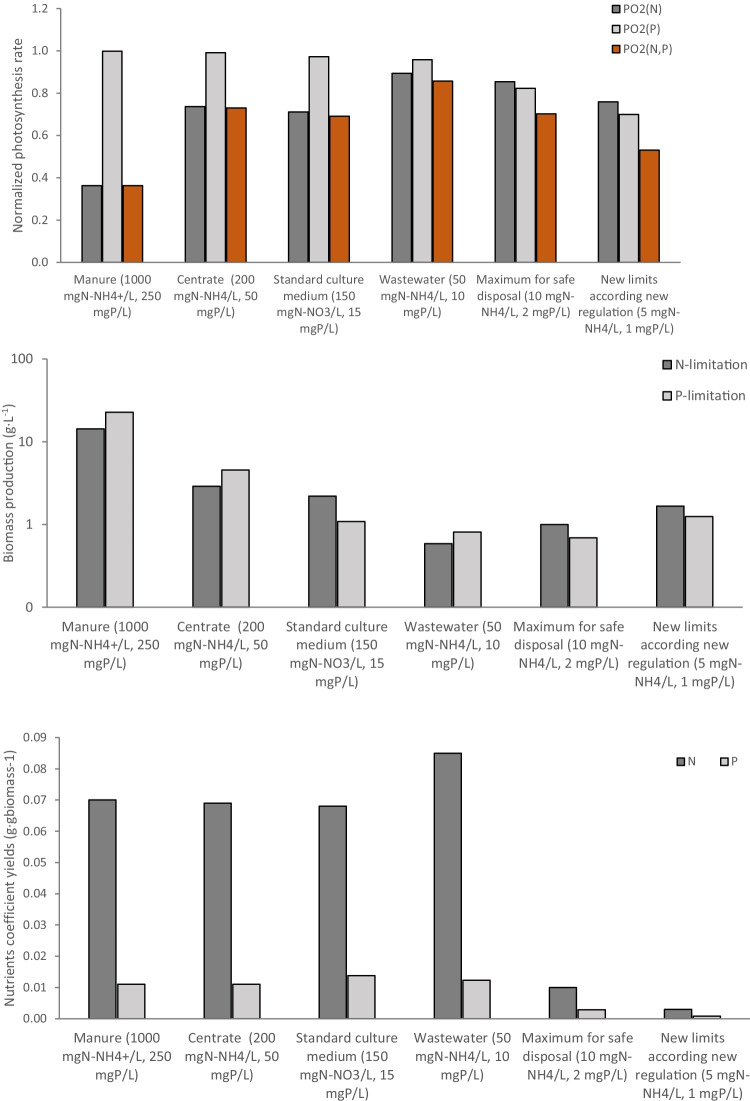
Simulations of the nitrogen and phosphorous effect in different culture media on the normalized photosynthesis rate (A), the nutrient yield coefficient (B), and biomass production (C)

Figure 6A shows the normalized photosynthesis rate as a function of the nitrogen and phosphorous concentration when using different culture media. Concerning nitrogen, the results shows that the normalized photosynthesis rate was maximal when using wastewater and wastewater after treatment, whereas it reduced because of nitrogen limitation when totally depurated wastewater was used. Conversely, when using manure or centrate as the culture medium, the photosynthesis rate decreased as a result of inhibition; this included fertilizers with high nitrogen concentrations. Regarding phosphorus, a different trend was observed. No inhibition was observed as a result of excess phosphorus, regardless of the culture medium used. A limitation in the photosynthesis rate only took place when totally depurated wastewater was used as the culture medium. Because the performance of the photosynthetic process is a function of both nitrogen and phosphorous availability, the performed simulations showed the photosynthesis rate of *S. almeriensis* decreased sharply when using manure or centrate as the culture medium. In contrast, *S. almeriensis* performed at its maximal capacity when using wastewater and treated wastewater as the culture medium.

The same scenarios were used to simulate the nutrient yield coefficients as a function of the nitrogen and phosphorus contained in the culture media (Fig. 6B). The results show that *S. almeriensis* consumed from 0.003 to 0.085 gN·g_biomass_^−1^, with maximal values being obtained when using wastewater and standard culture media, whereas both were reduced when excess or limiting concentrations of nitrogen were provided. The same behavior was observed for the phosphorous yield coefficients, which varied from 0.001 to 0.014 gP·g_biomass_^−1^, with maximal values also being obtained when using wastewater and standard culture medium.

Due to the diverse nutrient availability in the different culture media and the above-described variation in the yield coefficients as a function of nutrient availability, to calculate how much biomass can be produced per liter of culture medium for the different culture media is an interesting parameter (Fig. 6C). This analysis can be performed considering either N or P as the limiting nutrient, thus allowing us to identify which is the limiting factor when using the different culture media. The data shows that when using manure, up to 14.3 g of biomass can be produced per liter of manure, this production capacity being limited by the nitrogen concentration in the effluent, with the phosphorous content producing up to 22.7 g of biomass per liter. This biomass production capacity per liter of effluent was less for the other culture media. In the case of centrate, the maximal biomass production capacity was 2.9 g of biomass per liter, with nitrogen as the limiting nutrient. When using wastewater, the maximal biomass production capacity was 0.6 g of biomass per liter, again with the nitrogen concentration as the limiting factor. Also, phosphorous is the limiting nutrients when treated wastewater is used as a culture medium, so it is theoretically possible to produce 0.7 and 1.3 g of biomass per liter using treated wastewater with the maximum nutrient concentration for safe disposal and treated wastewater complying to the new limits, respectively.

## Discussion

Nitrate is the most convectional source of nitrogen used in microalgae cultures. In large-scale production systems, it is supplied in excess to avoid nutrient limitation (above 1000 mgNitrate·L^−1^, which corresponds to 225 mgN·L^−1^) (Acién et al. 2012). In the case of wastewaters, nitrogen mainly comes in the form of ammonium, with only minor concentrations of nitrate are detected when nitrification takes place, and always below 220 mgNitrate·L^−1^ (approximately 50 mgN·L^−1^). To study the influence of nitrate concentration on *Scenedesmus almeriensis* performance, concentrations ranging from 0 to 200 mgN·L^−1^ were assayed, which correspond to a nitrate range from 0 to 900 mgNitrate·L^−1^. By fitting experimental data to the Haldane equation, the nitrogen half-saturation constant (*K*_S,N-NO3_^−^ = 2.77 mgN-NO_3_^−^·L^−1^) and the inhibition parameter constant (*K*_I, N-NO3_^−^ = 279 mgN-NO_3_^−^·L^−1^) were determined. The nitrogen half saturation constant described for different *Scenedesmus* strains varies widely. An early kinetic model of *Scenedesmus dimosphus* growth and nutrient uptake proposed a nitrogen half-saturation constant of 0.018 mgN·L^−1^ using nitrate as the nitrogen source (Kunikane and Kaneko 1984), which is considerably less than that proposed in this work (K_S,N-NO3_^−^ = 2.77 mg· N-NO_3_^−^·L^−1^). In addition, recent research indicates the same variability with respect to the nutrient kinetic parameters. For instance, the nitrogen half-saturation constant obtained when *Scenedesmus* sp. is cultivated at different nitrate concentrations was 11.8 mgN·L^−1^. Furthermore, the authors did not observe microalgae growth inhibition as high nitrate concentration. However, it is important to note that no more than 25 mgN·L^−1^ was tested (Xin et al. 2010). Another previous work in which the nitrogen half-saturation constant was determined in an airlift-raceway reactor, using both *Scenedesmus* sp*.* and *Nannochloropsis salina*, showed a nitrogen half-saturation constant of 0.2 mgN·L^−1^ (Ketheesan and Nirmalakhandan 2013). Therefore, comparing the saturation coefficients collected in the bibliography together with the parameters determined in this study is especially difficult, since in each case a specific methodology (respirometric or through traditional tests), different nutrients and study times are applied. Concerning the respiration rate, the characteristic parameter values obtained were *K*_R, N-NO3_^−^ = 1.02 mgN-NO_3_^−^·L^−1^ and *K*_I,R,N-NO3_^−^ = 279 mg N-NO_3_^−^·L^−1^. The results show that the selected microalgae only need low nitrogen concentrations to perform the photosynthesis and respiration properly.

Regarding the influence of N-NH_4_^+^, this is the most frequent nitrogen source in wastewater, with concentrations ranging from 0 to 130 mg N-NH_4_^+^·L^−1^. It has been widely reported that N-NH_4_^+^ reduces the performance of microalgae cultures, especially at concentrations above 100 mgN·L^−1^ (approximately 130 mg N-NH_4_^+^·L^−1^) (Cabanelas et al. 2013). The results showed both the net photosynthesis rate and the net respiration rate increased along with the N-NH_4_^+^ concentration until a value of 10–20 mg N-NH_4_^+^ ·L^−1^ was reached; above this value, both the net photosynthesis rate and the net respiration rate decreased. These experimental data were fitted using the Haldane equation, in which the characteristic parameter values for the net photosynthesis rate (PO_2_) were determined (*K*_S,N-NH4_^+^  = 1.54 mgN-NH_4_^+^ L^−1^ and *K*_I, N-NH4_^+^  = 571 mgN-NH_4_^+^). Moreover, the kinetic parameters for the respiration rate (RO_2_) were *K*_R, N-NH4_^+^ = 0.65 mgN-NH_4_^+^·L^−1^ and *K*_I,R, N-NH4_^+^ = 205 mgN-NH_4_^+^·L^−1^. Despite the scarcity of nutrient half saturation constants obtained by respirometric tests, these results are comparable with a previous work in which the ammonia half-saturation constant for the Chlorophyta microalgae *Chlorella protothecoides* was determined (*K*_S,NH4_^+^ = 14.23 mgN-NH_4_^+^·L^−1^ (Sforza et al. 2019). The ammonia saturation coefficient described for *Chlorella protothecoides*, which was obtained using a similar respirometric protocol, was higher than for the same parameter in *S. almeriensis*. Furthermore, the respirometric experiments with *Chlorella protothecoides* did not show ammonia inhibition. However, the tests were performed in the 0–40 mgN-NH_4_^+^·L^−1^ range, which is significantly lower than the range tested here with *S. almeriensis*. The tests described in this work reached fairly high ammonia concentrations, which might explain the photosynthetic inhibitory effect. Rossi et al. (2020) used photo-respirometric tests to determine the EC_50,NH3_, which represents the free ammonia concentration causing a 50% inhibition of photosynthetic activity in a microalgae monoculture. They evaluated two *Scenedesmus* strains, *S.quadricuada* and *S.obliquus*, which showed an EC_50,NH3_ of 77.7 and 52.6 mgNH_3_·L^−1^, respectively (Rossi et al. 2020). At these concentrations, *S. almeriensis* showed a reduction in net photosynthesis of 20% and 10%, respectively, lower than that described for the other strains. However, the exposure time for *S. quadricuada* and *S. obliquus* was longer than that for *S. almeriensis*, which might have affected the results.

Apart from respirometric experiments, previous works have evaluated the influence of ammonia concentration on microalgae growth. These experiments founded that specific microalgae growth rate values showed no obvious differences to those in which the ammonia concentration was below 15–20 mgN-NH_4_^+^·L^−1^. However, when the free ammonia increased above 30–40 mgN-NH_4_^+^·L^−1^, the specific growth rate decreased. Compared to the optimal growth rate, the specific growth rate decreased by more than 50% and 80% when the free ammonia concentration increased to 30–40 mgN-NH_4_^+^·L^−1^ and 50–60 mgN-NH_4_^+^·L^−1^, respectively (Tan et al. 2016). These results showed an inhibitory effect at lower concentrations than those proposed in this work. Thus, it is essential to point out that the inhibitory effects seen in the short respirometric test could be aggravated if the test were longer.

As the data reported here show, *S. almeriensis* microalgae photosynthesize properly whether ammonium (or ammonia; note that they are in chemical equilibrium) or nitrate is used as the nitrogen source. The lab-scale experiments developed in this work have been performed using pure *S. almeriensis* cultures in which nitrate and ammonium have been tested separately. However, when microalgae are used to treat wastewater, both nitrate and ammonium appear as contaminants. To improve the microalgae wastewater treatment models, they should take into account that ammonium is generally preferred when both ammonium and nitrate are present (Mengesha et al. 1999; Solimeno et al. 2015).

Regarding phosphorus, this appears in the natural environment and wastewater in many forms such as orthophosphate (containing one phosphate unit), polyphosphate, pyrophosphate, metaphosphate, and their organic complexes. However, the main form from which microalgae acquire phosphorus is inorganic phosphate P-PO_4_^3−^ (orthophosphate) (Procházková et al. 2014; Khanzada 2020). Thus, most of the culture media reported for microalgae production contain phosphate in phosphorous form. The phosphorous concentration in regular microalgae culture media is much lower than the nitrogen concentration (up to ten times lower) whereas in some culture media, such as Arnon, it is even higher. In wastewater, the usual phosphorous concentration is much lower than the nitrogen concentration, with values ranging from 0 to 20 mg P-PO_4_^3−^·L^−1^ (Acién et al. 2016). Wastewaters coming from the mineral fertilizer industry can also contain high phosphorous concentrations, from 13 to 60 mg P-PO_4_^3−^·L^−1^ (Moreno Osorio et al. 2019). In this work, the experiments were performed at 120 mg PO_4_^3−^·L^−1^, which corresponds to 40 mg P-PO_4_^3−^·L^−1^. The characteristic parameter values for the net photosynthesis rate and the net respiration rate related to the phosphorous concentration were *K*_S,P-PO4_ = 0.43 mg P-PO_4_^3−^·L^−1^ and *K*_R, P-PO4_ = 0.35 mg P-PO_4_^3−^·L^−1^.

The phosphorous half-saturation constant obtained in the respirometric tests closely corresponds to the value obtained for of *Scenedesmus* sp. grown in batch mode in culture media modified with different phosphorous concentrations (*K*_S, P-PO4_ = 0.28 mg P-PO_4_^3−^·L^−1^) (Xin et al. 2010). However, these values are higher than those reported for *Scenedesmus obliquus*, which was studied in a mineral medium at different phosphorous and temperature values. The phosphorous half-saturation constant described ranged from 0.2 to 1.33 µM, which corresponds to 0.006 to 0.04 mg P-PO_4_^3−^·L^−1^ (Martínez et al. 1999). In addition, these authors reported growth inhibition at high phosphorous concentrations, which was not observed in this study. Despite most of the references revealing low phosphorous half-saturation coefficient values, a similar photo-respirometric work with *Chlorella protothecoides* showed a phosphorous half-saturation coefficient of 1.8 mg P-PO_4_^3−^·L^−1^. In short experiments, which take a few minutes, the observed effect of phosphorus on increased microalgae photosynthesis is due to phosphorous incorporation into the microalgal biomass, which could be used for metabolism (Sforza et al. 2019).

In respect of the yield coefficients; that is to say, how much of the nutrients are consumed from the culture medium per mass unit of already-produced biomass. Experiments were performed under the same concentration ranges as before, to determine if nutrient concentrations influence the coefficient yield values, as has previously been reported (Gómez-Serrano et al. 2015; Morales-Amaral et al. 2015). The data show that the nitrogen and phosphorous coefficient yields are not constant, being modified as a function of the nutrient’s concentration. The results show that the nitrogen and phosphorous coefficient yields increase as nitrogen or phosphorus increase in the culture medium. This variability in phosphorus uptake has already been previously described in a mixed microalgal consortium dominated by *Scenedesmus* at increasing phosphate concentrations. In practice, when phosphate aqueous concentration increased from 5 to 15 mgP-PO_4_^3−^·L^−1^, the microalgal acid soluble polyphosphate content increased up to three times (Powell et al. 2009). This phenomenon, by which microalgae cells are capable of taking up and storing more nutrients in larger amounts than necessary for immediate growth, is termed “luxury uptake” (Solovchenko et al. 2019). Apart from nutrients concentration, environmental variables such as temperature or light intensity may influence on luxury uptake of phosphorus by microalgae too (Powell et al. 2008).

Modelling this phenomenon is complex, since if the trend of the experimental data is considered, one might think that a certain inhibition appears in the yield coefficients. However, it would not be an inhibition, but the data show a variability in the value of the yield coefficients due to its relationship with the concentration of nitrogen and phosphorus in the medium. Regarding nitrogen, the coefficient values obtained ranged from 0.02 to 0.09 gN-NO_3_^−^·g_biomass_^−1^, which were in the same range as applied by Reichert et al. (2001) their mathematical models, with 0.065 gN·g_COD-ALG_^−1^ (Reichert and Vanrolleghem, 2001). Concerning the phosphorus, there are fewer references available in the literature related with phosphorus consumption by microalgae. The results showed that the phosphorous yield coefficient ranged from 0.004 to 0.014 gP-PO_4_^3−^·g_biomass_^−1^ at the phosphorous concentrations tested. Within this range, most of the previously described values in wastewater treatment appear (Reichert and Vanrolleghem 2001; Solimeno et al. 2017).

As previously explained for the kinetic parameters regarding the influence of nitrogen and phosphorous availability on the photosynthesis rate, the information found in the literature on the nitrogen and phosphorous coefficient yields is also highly variable. This may be due to the wide variety of microalgae strains and culture conditions tested. On the other hand, both the specific strain requirements and the methodology applied are complex and diverse.

Because the performance of the photosynthetic process is a function of both nitrogen and phosphorous availability, the performed simulations showed the photosynthesis rate of *S. almeriensis* decreased sharply when using manure or centrate as the culture medium. When using this strain to treat these effluents, great attention must be given to the effluent dosage in the reactor. In contrast, *S. almeriensis* performed at its maximal capacity when using wastewater and treated wastewater as the culture medium, making this strains’ application highly recommendable for wastewater treatment processes. The variation in the photosynthesis rate of *S. almeriensis* at different nutrients concentrations must be taken into account due to its influence on the oxygen production rate and related dissolved oxygen concentration, which determine the required mass transfer capacity and the overall design of the reactor.

Furthermore, an analysis was performed to determine how much biomass can be produced per liter of culture medium using the yield coefficients determined previously and the culture media proposed. The data showed that when using manure, up to 14.3 g of biomass can be produced per liter of manure considering the nitrogen concentration in the effluent and up to 22.7 g of biomass can be produced with the phosphorous content. Related to the other culture media such as centrate, it is possible to achieve 2.9 g of biomass per liter with nitrogen as the limiting nutrient. The use of wastewater with high contents in nitrogen as an ammonium form (manure or centrate), making it necessary to dilute this effluent prior to use as the culture medium inside the reactor to prevent to avoid inhibition caused by an excess of ammonium or others micropollutants, such as heavy metals, and because the color they have prevent light penetration (Acién et al. 2016; García et al. 2017). For that, knowing the exact composition of the wastewater to be treated is mandatory for an optimal treatment process and biomass production, not only to avoid inhibition processes but also to determine if additional carbon, nitrogen, or phosphorus need to be added when a low nutrient concentration appear. When using standard culture medium was phosphorus the limiting factor, but this could easily be corrected for by modifying its input into the culture medium, whereas modifying the effluent composition is a far more difficult matter. Also, phosphorous is the limiting nutrients when treated wastewater is used as a culture medium, being possible to produce 0.7 and 1.3 g of biomass per liter using treated wastewater with the maximum nutrient concentration for safe disposal and treated wastewater complying to the new limits, respectively.

In summary, results demonstrated that the photosynthesis rate and the respiration rate of *Scenedesmus almeriensis* vary as a function of nutrient availability (N-NO_3_^−^, N-NH_4_^+^, and P-PO_4_^3−^). Regarding nitrogen, both in the form of N-NO_3_^−^ and N-NH_4_^+^, a similar trend was observed with inhibition taking place at high concentrations, whereas no inhibition by phosphorous was observed. Regarding the nutrient yield coefficients, data show that the luxury uptake phenomenon appears at increasing nutrient concentrations, while above a limit, the nutrient yield coefficients remain constant. Both the photosynthesis/respiration rates and the nutrient yield coefficients have been modelled as a function of nutrient availability in the medium. To the best of our knowledge, this is the first time that such models have been proposed, including the luxury uptake phenomenon in microalgae cultures. These results highlight the importance of the concentration of nutrients in the microalgae culture, which is a decisive factor together with operational factors such as the pH of the culture or the temperature. With the aim of working in the most optimal conditions possible since it is crucial to achieve the maximum performance in microalgae cultures. These models must be considered in microalgae-related systems in order to optimize them, whether using inorganic fertilizers or wastewater. In the former, it is necessary to optimize the culture medium composition according to the system performance and nutrient demand. In the latter, the challenge is to determine the optimal conditions for maximizing the nutrient removal and biomass production capacity because the wastewater composition cannot be modified.

## Data Availability

The data that support the findings of this study are available from the corresponding author on request.
